# Recurrence timing and visual outcomes of recurrent myopic choroidal neovascularization after anti-VEGF therapy

**DOI:** 10.1186/s40942-026-00900-8

**Published:** 2026-07-11

**Authors:** Nala Shin, Ki Young Son

**Affiliations:** 1 Department of Ophthalmology, Chungnam National University Hospital, Daejeon, Republic of Korea; 2 Department of Ophthalmology, Chungnam National University Sejong Hospital, Sejong, Republic of Korea; 3 Department of Digital Health, Samsung Advanced Institute for Health Sciences and Technology (SAIHST), Sungkyunkwan University, Seoul, Republic of Korea

**Keywords:** Myopia, Choroidal neovascularization, Recurrence, Visual acuity, Optical coherence tomography

## Abstract

**Purpose:**

To compare clinical characteristics and visual outcomes according to recurrence timing in eyes with myopic choroidal neovascularization (CNV) following anti-vascular endothelial growth factor (anti-VEGF) therapy.

**Methods:**

This retrospective cohort study included eyes with treatment-naïve myopic CNV that achieved complete resolution after initial anti-VEGF therapy. To specifically evaluate the impact of recurrence timing, analyses were restricted to eyes that developed recurrence, which were categorized as early (< 12 months) or late (≥ 12 months). Baseline characteristics, multimodal imaging features, treatment profiles, and recurrence-specific parameters were compared. Longitudinal changes in best-corrected visual acuity (BCVA) were analyzed using linear mixed-effects models, and factors associated with the final BCVA were evaluated using multivariable linear regression.

**Results:**

Of the 79 eyes, 32 (40.5%) experienced recurrence during the mean follow-up of 36.9 ± 23.7 months (early: 15 eyes; late: 17 eyes). The early recurrence group exhibited a significantly longer axial length (*p* = 0.019); other baseline features and treatment profiles were similar between groups. At recurrence, subretinal hyperreflective material height was significantly greater in the late recurrence group (*p* = 0.041). Both groups showed visual improvement after initial treatment, without a significant difference in BCVA at any predefined time point. Baseline BCVA was the only independent predictor for final BCVA (β = 0.46, *p* = 0.039); no significant association between recurrence timing and visual outcomes was detected.

**Conclusion:**

Visual outcomes were comparable between early and late recurrences of myopic CNV. Baseline BCVA was the strongest factor associated with long-term visual outcome, whereas no association between recurrence timing and visual outcomes was detected in this cohort.

## Key message

• Myopic choroidal neovascularization (CNV) frequently recurs after anti-VEGF therapy, and several clinical and imaging factors associated with recurrence have been identified. Previous studies have also shown that baseline visual acuity is a key determinant of long-term visual outcomes.

• This study specifically evaluates the clinical significance of recurrence timing in eyes with recurrent myopic CNV. By restricting the analysis to eyes that developed recurrence, we demonstrate that although early and late recurrence differ in clinical and structural characteristics, no association between recurrence timing and visual outcomes was detected in this cohort.

• These findings may help refine follow-up strategies and improve patient counseling in clinical practice, while highlighting the importance of continuous monitoring and timely retreatment regardless of when recurrence occurs.

## Introduction

Myopic choroidal neovascularization (CNV) is a complication of pathological myopia that can lead to substantial loss of central vision, particularly in working-age individuals [1]. Previous epidemiological studies have estimated that myopic CNV develops in approximately 5–11% of highly myopic eyes, often resulting in progressive central vision loss if left untreated [2]. 

Intravitreal anti-vascular endothelial growth factor (anti-VEGF) therapy has significantly improved the visual outcomes in individuals with myopic CNV; most eyes demonstrate rapid anatomical resolution and visual improvement after a limited number of injections [3–8]. However, despite favorable initial responses, recurrence remains a significant clinical challenge. Previous studies have reported recurrence rates ranging from approximately 23% to over 30% within the first 1–2 years, and a persistent risk of reactivation during longer follow-up periods [9–12]. 

Several clinical and imaging factors have been associated with myopic CNV recurrence, including larger CNV size, greater initial treatment burden, and structural disruptions, such as ellipsoid zone and retinal pigment epithelium (RPE) damage [13, 14]. These findings suggest that recurrence is closely related to the underlying disease activity and pre-existing structural damage. Importantly, previous studies have shown that recurrence does not necessarily lead to worse long-term visual outcomes when timely retreatment is provided, and baseline visual acuity remains the strongest predictor of final visual prognosis [9, 15, 16]. 

Although prior research has primarily focused on identifying recurrence risk factors, the clinical significance of recurrence timing among eyes in which recurrence has already occurred has not been well elucidated. In particular, whether early recurrence indicates a more aggressive disease phenotype or has a different visual prognosis compared to late recurrence remains unclear.

Understanding these temporal patterns is crucial for optimizing follow-up strategies and managing patient expectations. Accordingly, this study evaluated the clinical significance of recurrence timing by comparing visual outcomes and clinical characteristics between early and late recurrences in eyes with recurrent myopic CNV after anti-VEGF therapy.

## Materials and methods

### Study design and participants

This retrospective cohort study was conducted at two tertiary referral hospitals in Korea: Chungnam National University Hospital and Chungnam National University Sejong Hospital. This study adhered to the tenets of the Declaration of Helsinki and was approved by the Institutional Review Board of Chungnam National University Sejong Hospital (IRB No. 2025-12-010). The requirement for informed consent was waived due to the retrospective design of the study and anonymized data.

Patients diagnosed with treatment-naïve myopic CNV who received intravitreal anti-VEGF therapy between January 2017 and December 2024 were included.

The inclusion criteria were: (1) pathologic myopia defined as axial length ≥ 26.5 mm and/or spherical equivalent ≤ − 6.0 diopters; (2) presence of active CNV secondary to pathologic myopia confirmed by spectral-domain optical coherence tomography (OCT) and fluorescein angiography (FA), with or without indocyanine green angiography; and (3) minimum follow-up duration of 12 months after initial CNV inactivation.

The exclusion criteria were: (1) presence of other macular diseases, including a macular hole or CNV associated with age-related macular degeneration or inflammatory causes; (2) severe myopic traction maculopathy (MTM) or retinal detachment; (3) prior pars plana vitrectomy or scleral buckling surgery; and (4) prior treatment with intravitreal injections or photodynamic therapy.

The eyes with recurrence were categorized into early (< 12 months) and late (≥ 12 months) recurrence groups based on the interval from initial resolution to recurrence. The 12-month cutoff was selected because previous studies have shown that most recurrences of myopic CNV occur within the first year after treatment, making this clinically meaningful threshold for distinguishing early and late recurrence [6, 12]. The comparative analyses were restricted to eyes that experienced recurrence.

### Ophthalmic examinations and imaging analysis

All patients underwent a comprehensive ophthalmologic evaluation before treatment initiation that included best-corrected visual acuity (BCVA), refraction, slit-lamp examination, dilated fundus examination, and intraocular pressure measured using a non-contact tonometer (CT-800 A, Topcon Healthcare Corporation, Tokyo, Japan). Axial length was measured using IOLMaster (Carl Zeiss Meditec, Jena, Germany).

Multimodal imaging included color fundus photography (Topcon TRC-NW8; Topcon Corporation), spectral-domain OCT (Cirrus HD-OCT 5000, Carl Zeiss Meditec), fluorescein angiography, and indocyanine green angiography when available (Heidelberg Retina Angiograph, Heidelberg Engineering, Heidelberg, Germany). Indocyanine green angiography was performed in 72 of 79 eyes (91.1%) in the overall cohort.

Myopic CNV was diagnosed based on multimodal imaging findings, including early hyperfluorescence with late leakage on FA and corresponding intra- or subretinal fluid on OCT. CNV lesion size was measured on early-phase FA images using manual delineation, and lesion location was classified as subfoveal, juxtafoveal, or extrafoveal, with non-subfoveal lesions defined as ≥ 200 μm from the foveal center.

The OCT parameters, including subfoveal choroidal thickness, CNV height and width, and subretinal hyperreflective material (SHRM) dimensions, were measured using calipers. Central retinal thickness was automatically calculated as the mean thickness within the central 1-mm Early Treatment Diabetic Retinopathy Study subfield. Only scans with a signal strength ≥ 7 and without segmentation errors were included.

The baseline structural features were evaluated using OCT. Ellipsoid zone integrity was classified as intact, partially disrupted, or disrupted, and RPE status was classified as intact, irregular, or atrophic. MTM was graded as: stage 0, no MTM; stage 1, epiretinal membrane or vitreomacular traction without retinoschisis; stage 2, foveoschisis or lamellar macular hole; and stage 3, full-thickness macular hole or macular detachment.

All imaging assessments were independently evaluated by two blinded graders (NS and KYS), and discrepancies were resolved by consensus.

### Treatment protocol and follow-up

All eyes received intravitreal anti-VEGF injections at baseline. The treating ophthalmologist selected one of the following agents: bevacizumab (1.25 mg), ranibizumab (0.5 mg), or aflibercept (2 mg). The initial regimen consisted of three consecutive monthly injections, followed by a pro re nata regimen.

At each follow-up visit, disease activity was assessed using BCVA, fundus examination, and OCT. Eyes were retreated for the presence of intra- or subretinal fluid, SHRM, new or persistent hemorrhage, or visual deterioration attributable to CNV activity.

Initial resolution was defined as the absence of intra- and subretinal fluid on OCT with stabilization or improvement in BCVA. The time to resolution was calculated from the first injection to the first visit meeting these criteria. After initial resolution, the patients were followed up at 1–3 month intervals.

Recurrence was defined as reactivation of CNV after documented resolution, as indicated by new or increased intra- or subretinal fluid on OCT or leakage on FA. To clearly distinguish true recurrence from persistent disease activity, recurrence was strictly defined as the reactivation of CNV occurring at least 6 months after the last injection, following a documented period of resolution.

### Outcome measures and statistical analyses

The primary analysis focused on eyes with recurrence. The primary outcome was the final BCVA at the last follow-up visit, interpreted in the context of follow-up duration. The secondary outcomes included BCVA at predefined follow-up time points to enable standardized comparisons, longitudinal changes in BCVA, and recurrence characteristics.

The continuous variables are presented as medians with interquartile ranges, and categorical variables are presented as numbers (percentages). Between-group comparisons were performed using the Mann–Whitney U test for continuous variables and the χ² test or Fisher’s exact test for categorical variables, as appropriate.

Longitudinal changes in BCVA were analyzed using linear mixed-effects models to account for correlations among repeated measurements within the same eye. A multivariable linear regression analysis was performed to identify factors associated with the final BCVA. The model included recurrence timing, axial length, baseline BCVA, and baseline intraretinal fluid (IRF) as covariates. To account for potential bias due to differences in follow-up duration between the groups, the total follow-up duration was additionally included as a covariate in the final model. As an exploratory analysis, time to recurrence was also evaluated as a continuous variable to assess its association with final BCVA. Significance was defined as a two-sided p value < 0.05. All analyses were performed using R software (R Foundation for Statistical Computing, Vienna, Austria).

## Results

### Study population and baseline characteristics

**Fig. 1:**
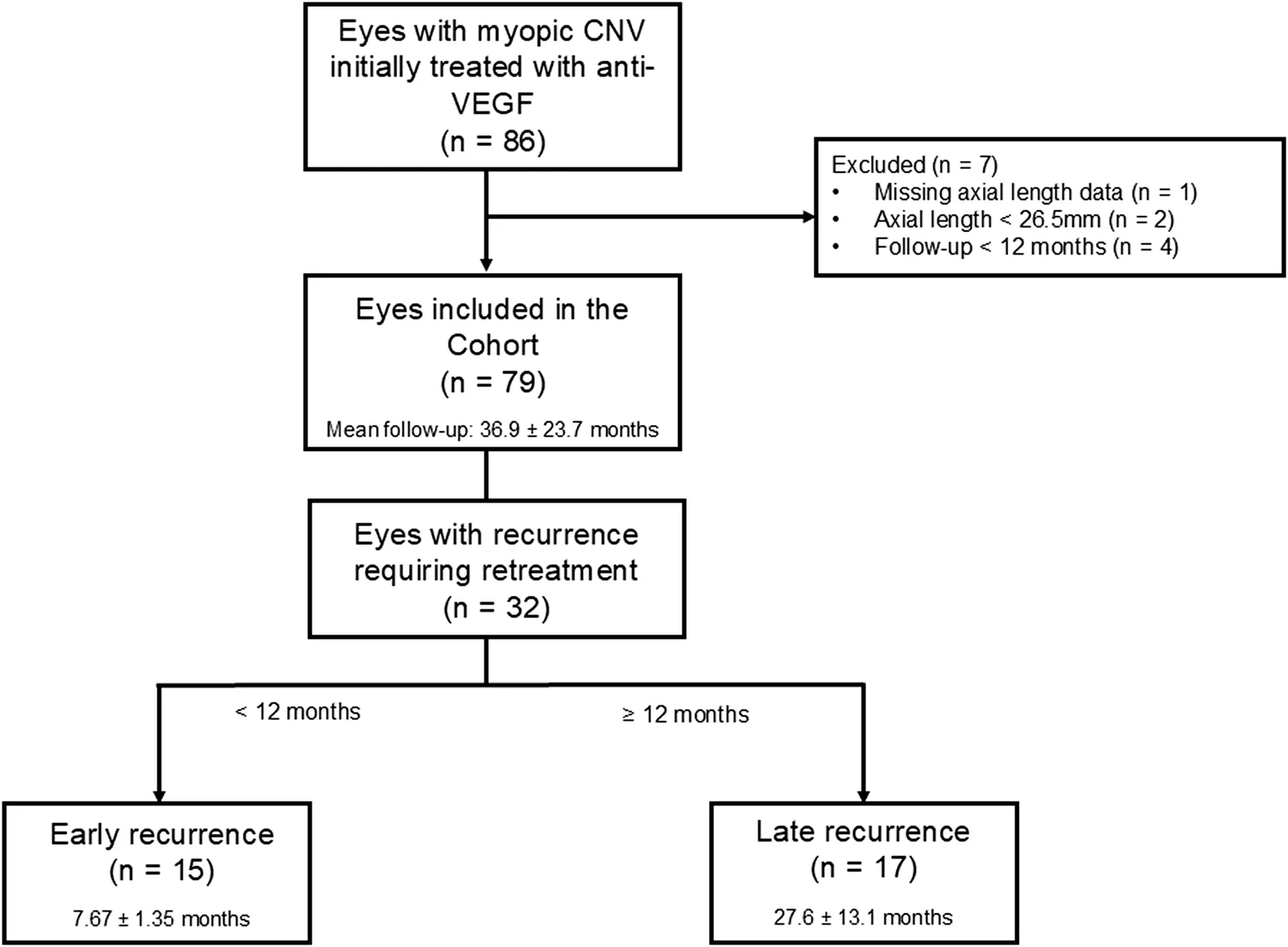
Study flow chart. Flowchart illustrating the selection of eyes with myopic choroidal neovascularization (CNV) treated with anti-vascular endothelial growth factor (anti-VEGF) therapy. Among the 86 eyes initially identified, 79 were included in the final cohort after applying the exclusion criteria. During follow-up, 32 eyes developed recurrences that required retreatment; they were classified into early (< 12 months, *n* = 15) and late (≥ 12 months, *n* = 17) recurrence groups based on the interval from initial resolution to recurrence. Subsequent comparative analyses were performed between the early and late recurrence groups. The values represent the mean ± standard deviation for time to recurrence

A total of 86 eyes with treatment-naïve myopic CNV were initially identified. After excluding seven eyes owing to missing data or unmet inclusion criteria, 79 eyes from 79 patients were included in the study cohort (Fig. 1). The mean follow-up duration for the entire cohort was 36.9 ± 23.7 months.

During follow-up, 32 eyes (40.5%) developed recurrence requiring retreatment. Subsequent analyses focused on these 32 eyes with recurrence. Among them, 15 eyes (46.9%) were classified into the early recurrence group (< 12 months) and 17 eyes (53.1%) into the late recurrence group (≥ 12 months).

**Fig. 2:**
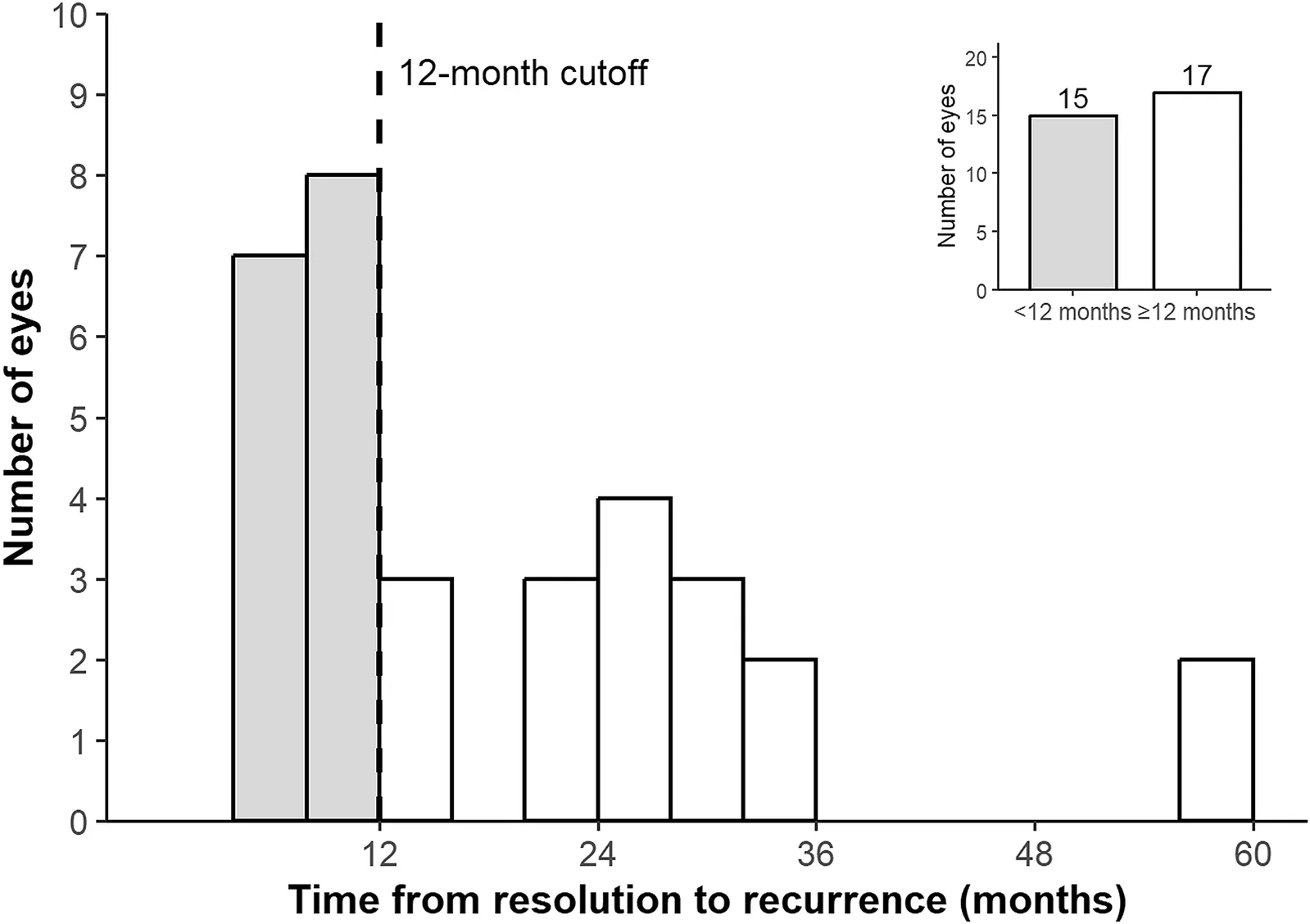
Distribution of recurrence time. Histogram showing the time distribution from initial myopic choroidal neovascularization (CNV) resolution to recurrence. The vertical dashed line indicates the 12-month cutoff used to define the early and late recurrence groups. The distribution suggests a bimodal tendency, with clusters of recurrence occurring within 12 months and after 24 months

The mean time from initial resolution to recurrence was 18.3 ± 13.8 months overall, and 7.67 ± 1.35 months in the early group and 27.6 ± 13.1 months in the late group. Figure 2 demonstrates a broad distribution of recurrence timing, with recurrence events occurring both within the first year after resolution and during longer-term follow-up extending beyond 24 months.

The baseline characteristics are summarized in Table 1. Notably, the participants in the early recurrence group exhibited a significantly longer axial length in the CNV eye than those in the late recurrence group (29.8 [29.3–30.5] mm vs. 28.7 [27.6–29.4] mm, *p* = 0.019). Additionally, the total follow-up duration was significantly shorter in the early recurrence group (30.0 [22.0–55.0] months vs. 60.0 [36.0–72.0] months, *p* = 0.028). Other factors, including age and baseline BCVA (0.40 [0.26–0.76] vs. 0.30 [0.30–0.70] logMAR, *p* = 0.774), and systemic comorbidities were not significantly different.

**Table 1:** Baseline characteristics of eyes with early and late recurrence of myopic choroidal neovascularization after Anti-VEGF therapy

Variable	Early recurrence (15 eyes)	Late recurrence (17 eyes)	*P* value
Age, years	56.0 [38.0, 68.5]	61.0 [57.0, 72.0]	0.140
Female, n (%)	14 (93.3)	16 (94.1)	1.000
Laterality, Left, n (%)	7 (46.7)	7 (41.2)	1.000
Diabetes mellitus, n (%)	0 (0.0)	1 (5.9)	1.000
Hypertension, n (%)	4 (26.7)	5 (29.4)	1.000
Intraocular pressure, mmHg	17.0 [15.5, 19.0]	16.0 [15.0, 18.0]	0.435
Axial length (CNV eye), mm	29.8 [29.3, 30.5]	28.7 [27.6, 29.4]	**0.019**
Axial length (fellow eye), mm	29.2 [28.3, 30.2]	28.3 [27.0, 29.0]	0.147
Pseudophakia, n (%)	6 (40.0)	8 (47.1)	0.964
Baseline BCVA (logMAR)	0.40 [0.26, 0.76]	0.30 [0.30, 0.70]	0.774
Previous ocular surgery, n (%)	11 (73.3)	10 (58.8)	0.625
Total follow-up, months	30.0 [22.0, 55.0]	60.0 [36.0, 72.0]	**0.028**

- Data are presented as median [interquartile range] or n (%)

- BCVA, best-corrected visual acuity; CNV, choroidal neovascularization; logMAR, logarithm of the minimum Angle of Resolution

### Ophthalmic imaging and recurrence characteristics

The baseline multimodal imaging findings are presented in Table 2. Most parameters, including CNV location (*p* = 0.406), size (*p* = 0.736), and subfoveal choroidal thickness (*p* = 0.601), were comparable between the groups. IRF at baseline was more frequently observed in the early recurrence group (46.7% vs. 11.8%), although the difference was not significant (*p* = 0.072).

**Table 2:** Baseline ophthalmic imaging characteristics of eyes with myopic choroidal neovascularization according to recurrence timing

Variable	Early recurrence (15 eyes)	Late recurrence (17 eyes)	*P* value
Lacquer cracks, n (%)	1 (6.7)	5 (29.4)	0.234
Patchy atrophy, n (%)	3 (20.0)	5 (29.4)	0.838
Posterior staphyloma, n (%)	4 (26.7)	6 (35.3)	0.886
Pathologic myopia grade, n (%)			0.874
grade 0	1 (6.7)	2 (11.8)	
grade 1	5 (33.3)	5 (29.4)	
grade 2	6 (40.0)	8 (47.1)	
grade 3	3 (20.0)	2 (11.8)	
grade 4	0 (0.0)	0 (0.0)	
Fuchs spot, n (%)	1 (6.7)	3 (17.6)	0.688
CNV location, n(%)			0.406
Subfoveal	6 (42.9)	7 (41.2)	
Juxtafoveal	4 (28.6)	2 (11.8)	
Extrafoveal	4 (28.6)	8 (47.1)	
CNV area (mm^2^)	0.24 [0.16, 0.43]	0.28 [0.19, 0.37]	0.736
CNV type, type2, n (%)	10 (71.4)	14 (82.4)	0.770
Subfoveal choroidal thickness, µm	95.0 [82.5, 110.0]	100.0 [90.0, 130.0]	0.601
Central subfield thickness, µm	301.0 [285.3, 342.8]	339.0 [287.0, 350.0]	0.416
CNV height, µm	110.0 [92.5, 150.0]	110.0 [100.0, 140.0]	0.984
CNV width, µm	615.0 [300.0, 707.5]	390.0 [310.0, 790.0]	0.953
SHRM height, µm	100.0 [82.5, 110.0]	90.0 [80.0, 100.0]	0.496
SHRM width, µm	745.0 [537.5, 835.0]	710.0 [400.0, 880.0]	0.721
Subretinal fluid, n (%)	5 (33.3)	7 (41.2)	0.927
Intraretinal fluid, n (%)	7 (46.7)	2 (11.8)	0.072
Subretinal hemorrhage, n (%)	9 (60.0)	7 (41.2)	0.479
EZ integrity, n (%)			0.645
Intact	1 (6.7)	3 (17.6)	
Partial disruption	10 (66.7)	10 (58.8)	
Disrupted	4 (26.7)	4 (23.5)	
RPE status, n (%)			0.376
Intact	5 (33.3)	9 (52.9)	
Irregular	9 (60.0)	6 (35.3)	
Atrophy	1 (6.7)	2 (11.8)	

- Data are presented as median [interquartile range] or number (%). CNV location and CNV area were determined using fluorescein angiography (FA) and indocyanine green angiography (ICGA). CNV location was categorized as subfoveal, juxtafoveal, or extrafoveal; juxtafoveal CNV was defined as a lesion margin within 200 μm of the foveal center, and extrafoveal CNV as a lesion margin located ≥ 200 μm from the foveal center. EZ integrity was classified as intact, partial disruption, or disrupted, and RPE status as intact, irregular, or atrophic. Continuous variables were compared using the Mann–Whitney U test, and categorical variables were compared using Fisher’s exact test or the χ² test, as appropriate

- FA, fluorescein angiography; ICGA, indocyanine green angiography; CNV, choroidal neovascularization; SHRM, subretinal hyperreflective material; SRF, subretinal fluid; IRF, intraretinal fluid; EZ, ellipsoid zone; RPE, retinal pigment epithelium

The treatment outcomes and recurrence features are summarized in Table 3. The number of initial injections (median 3.0 in both groups) and the time to initial resolution (8.0 weeks in both groups) were similar. However, at the time of recurrence, the late recurrence group demonstrated a significantly greater SHRM height (110.0 [80.0–160.0] µm vs. 70.0 [50.0–100.0] µm, *p* = 0.041), whereas other structural parameters remained similar between groups. Although aflibercept was used in one eye in the overall cohort, no aflibercept-treated eyes were included among the recurrent eyes analyzed in this study.

**Table 3:** Treatment outcomes and recurrence characteristics of eyes with myopic choroidal neovascularization according to recurrence timing

Variable	Early recurrence (15 eyes)	Late recurrence (17 eyes)	*P* value
**Treatment Characteristics**			
Symptom duration, days	14.0 [4.75, 30.0]	22.0 [6.5, 30.0]	0.815
Anti-VEGF type, n (%)			1.000
Bevacizumab	13 (86.7)	14 (82.4)	
Ranibizumab	2 (13.3)	3 (17.6)	
Number of initial injections	3.0 [3.0, 3.0]	3.0 [2.0, 4.0]	0.934
Total number of injections	6.0 [4.5, 8.5]	5.0 [4.0, 7.0]	0.391
Mean initial injection interval, weeks	4.0 [4.0, 4.0]	4.0 [4.0, 4.8]	0.762
Time to initial resolution, weeks	8.0 [4.0, 8.0]	8.0 [8.0, 12.0]	0.075
**Recurrence characteristics**			
Time from resolution to recurrence, months	8.0 [6.5, 9.0]	25.0 [21.0, 31.0]	**< 0.001**
Total number of recurrence episodes	1.0 [1.0, 2.0]	1.0 [1.0, 1.0]	0.485
Type of recurrence			0.321
Reactivation of original lesion	10 (66.7)	9 (52.9)	
New lesion development	4 (26.7)	8 (47.1)	
Mixed type	1 (6.7)	0 (0.0)	
CNV height, µm	120.0 [80.0, 140.0]	115.0 [80.0, 172.5]	0.758
CNV width, µm	460.0 [430.0, 580.0]	570.0 [372.5, 850.0]	0.345
SHRM height, µm	70.0 [50.0, 100.0]	110.0 [80.0, 160.0]	**0.041**
SHRM width, µm	430.0 [350.0, 930.0]	645.0 [475.0, 852.5]	0.417
Subretinal fibrosis, n (%)	0 (0.0)	2 (11.8%)	0.486

- Data are presented as median [interquartile range] or number (%). Continuous variables were compared using the Mann–Whitney U test, and categorical variables were compared using Fisher’s exact test. Resolution was defined as the absence of exudative signs after anti–vascular endothelial growth factor (anti-VEGF) treatment

- Anti-VEGF, anti–vascular endothelial growth factor; BCVA, best-corrected visual acuity; CNV, choroidal neovascularization; IQR, interquartile range; logMAR, logarithm of the minimum angle of resolution; SHRM, subretinal hyperreflective material

### Visual outcomes and predictive factors

**Fig. 3:**
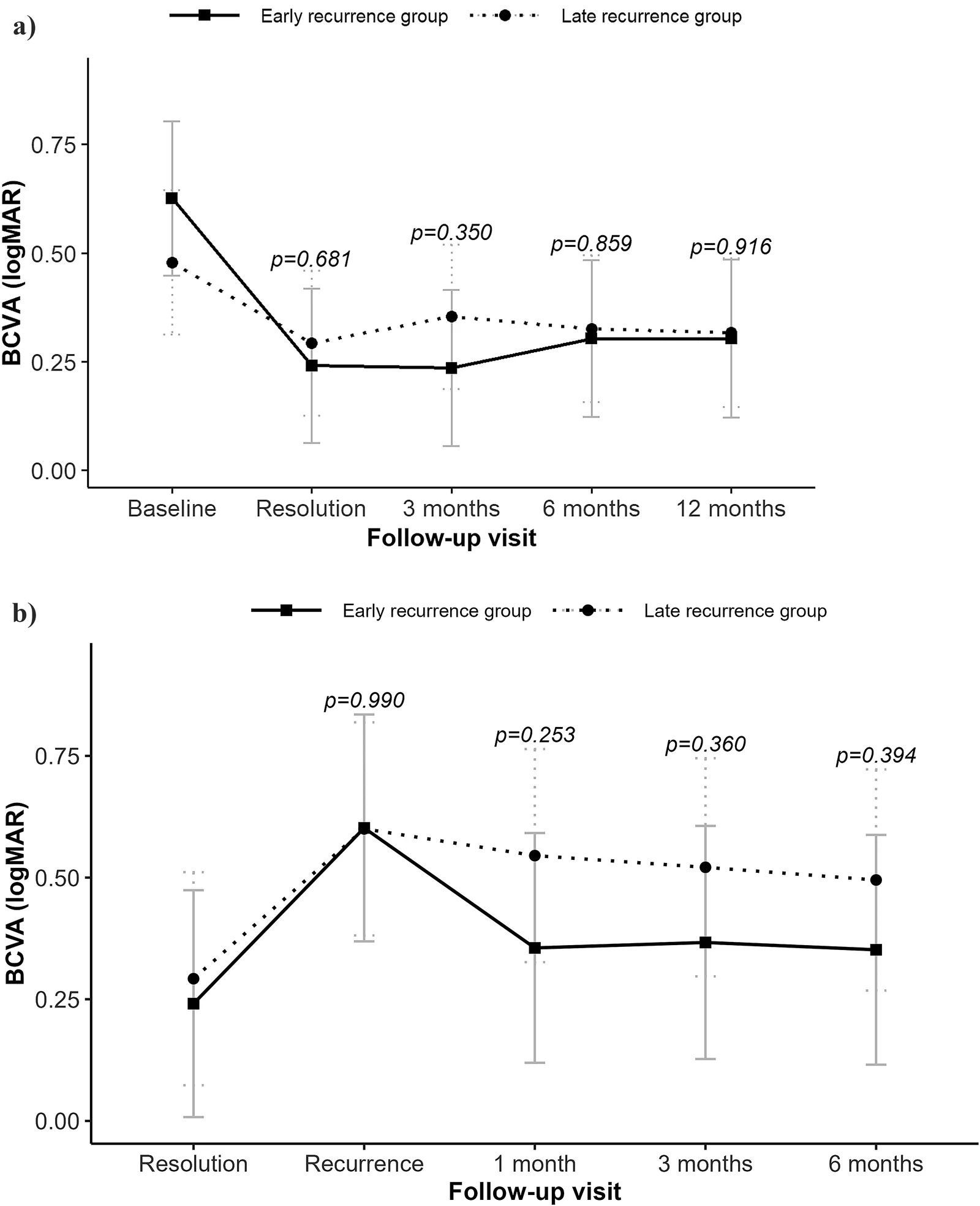
Longitudinal visual outcomes. **(a)** Resolution-based trajectory of best-corrected visual acuity (BCVA) from baseline to 12 months after initial resolution. BCVA improved after initial anti-vascular endothelial growth factor (anti-VEGF) treatment and remained stable during follow-up, without significant differences between the early and late recurrence groups at any time point. Numbers of eyes contributing BCVA measurements at each time point were as follows: early recurrence group, 15 at baseline, 15 at resolution, 14 at 3 months, 14 at 6 months, and 13 at 12 months; late recurrence group, 17 at baseline, 17 at resolution, 17 at 3 months, 16 at 6 months, and 15 at 12 months. Since recurrence occurred within 12 months in the early recurrence group, the 12-month BCVA measurements may reflect post-recurrence and post-retreatment outcomes.**(b)** Recurrence-based trajectory of BCVA from initial resolution to recurrence and up to 6 months after recurrence. BCVA worsened at the time of recurrence and subsequently improved following retreatment, without significant differences between groups at any time point. Numbers of eyes contributing BCVA measurements at each time point were as follows: early recurrence group, 15 at recurrence, 14 at 1 month, 13 at 3 months, and 14 at 6 months; late recurrence group, 17 at recurrence, 17 at 1 month, 15 at 3 months, and 14 at 6 months.The plotted values represent estimated marginal means derived from linear mixed-effects models. Error bars indicate 95% confidence intervals. The p values indicate between-group comparisons at each time point

The visual outcome details of the groups are presented in Table 4. Both groups had significant improvement after the initial anti-VEGF therapy (early recurrence group, *p* < 0.001; late recurrence group, *p* = 0.004). In the resolution-based trajectory analysis (Fig. 3a), both groups maintained improved BCVA compared with baseline, without significant differences between groups at any predefined time point (all *p* > 0.1).

**Table 4:** Visual outcomes during initial treatment and after recurrence according to recurrence timing

Variable	Early recurrence (15 eyes)	Late recurrence (17 eyes)	*P* value
**Visual Outcomes during Initial Treatment**			
Baseline BCVA (logMAR)	0.40 [0.26, 0.76]	0.30 [0.30, 0.70]	0.774
BCVA after initial resolution	0.20 [0.05, 0.30]	0.22 [0.05, 0.40]	0.703
BCVA at 3 months after resolution	0.15 [0.05, 0.21]	0.30 [0.10, 0.49]	0.194
BCVA at 6 months after resolution	0.20 [0.06, 0.62]	0.30 [0.14, 0.42]	0.676
BCVA at 12 months after resolution	0.22 [0.10, 0.70]	0.22 [0.10, 0.40]	0.745
**Visual Outcomes after Recurrence**			
BCVA at recurrence (logMAR)	0.30 [0.22, 0.91]	0.52 [0.30, 0.70]	0.985
BCVA at 1 month after recurrence	0.26 [0.02, 0.62]	0.40 [0.30, 0.70]	0.288
BCVA at 3 months after recurrence	0.22 [0.15, 0.82]	0.30 [0.19, 0.52]	0.547
BCVA at 6 months after recurrence	0.21 [0.06, 0.49]	0.35 [0.09, 0.40]	0.578
Final BCVA	0.22 [0.10, 0.46]	0.22 [0.10, 0.52]	0.849

- Data are presented as median [interquartile range]. Continuous variables were compared between groups using the Mann–Whitney U test

**-** BCVA, best-corrected visual acuity; CNV, choroidal neovascularization; logMAR, logarithm of the minimum angle of resolution

After recurrence, the recurrence-based trajectory analysis (Fig. 3b) showed that BCVA worsened transiently and recovered after retreatment. Significant differences in BCVA were not observed between the groups at the time of recurrence (*p* = 0.985) or at 1, 3, and 6 months after recurrence. No significant difference in final BCVA was detected between the groups at the last follow-up visit (0.22 [0.10–0.46] vs. 0.22 [0.10–0.52], *p* = 0.849).

The multivariable linear regression analysis (Table 5) identified baseline BCVA as the only independent predictor for final BCVA in eyes with recurrence (β = 0.46, 95% confidence interval: 0.04–0.88, *p* = 0.039). Recurrence timing (*p* = 0.258), axial length (*p* = 0.399), baseline IRF (*p* = 0.968), and follow-up duration (*p* = 0.419) were not significantly associated with final BCVA. As an exploratory analysis, time to recurrence was also evaluated as a continuous variable. In both univariable and multivariable linear regression analyses, time to recurrence was not significantly associated with final BCVA (univariable: β = 0.005, *p* = 0.436; multivariable: β = 0.011, *p* = 0.158).

**Table 5:** Multivariable linear regression analysis for factors associated with final visual outcome

Predictor	Estimate (β)	95% CI	*P* value
Recurrence timing (Late vs. Early)	0.27	-0.20 to 0.73	0.258
Axial length	0.07	-0.09 to 0.22	0.399
Baseline BCVA (logMAR)	0.46	0.04 to 0.88	**0.039**
Baseline IRF	0.01	-0.46 to 0.48	0.968
Total Follow-up duration (months)	-0.003	-0.01 to 0.005	0.419

- Data are presented as regression coefficients (β) with 95% confidence intervals. Multivariable linear regression analysis included recurrence timing group, axial length, baseline BCVA, baseline intraretinal fluid, and total follow-up duration as covariates

- BCVA, best-corrected visual acuity; IRF, intraretinal fluid; logMAR, logarithm of the minimum angle of resolution

## Discussion

In this study, we investigated the clinical significance of recurrence timing in eyes with myopic CNV that recurred after anti-VEGF therapy. Our analyses were restricted to eyes with recurrence, which allowed direct comparison of visual outcomes and clinical characteristics between the early and late recurrence groups. Although the early and late recurrence groups exhibited differences in baseline characteristics (axial length) and structural features at recurrence (SHRM height), the visual outcomes at the predefined follow-up time points were comparable between the groups. Furthermore, the multivariable analysis showed that no association between recurrence timing and final BCVA was detected in this cohort, even after adjustment for follow-up duration.

Myopic CNV recurrence is commonly observed after anti-VEGF therapy. Previous studies have reported recurrence rates of approximately 23–33% within the first 1–2 years [9–12]. In our cohort, recurrence occurred in 40.5% of eyes during a mean follow-up of 36.9 months, within the range reported in studies with extended follow-up. Notably, Borrelli et al. reported a 62.5% recurrence rate within 36 months, with a mean time to recurrence of 18.8 ± 15.3 months, which is comparable to our finding (18.3 ± 13.8 months) [17]. However, the discrepancy in recurrence rates may be attributed to differences in the definition of recurrence. In the present study, recurrence was defined using a more conservative criterion, requiring reactivation to occur at least 6 months after the last anti-VEGF injection following initial resolution. In contrast, previous studies have often defined recurrence based on exudation observed as early as 3 months after resolution [12, 18, 19]. 

One notable finding of this study was the temporal distribution of recurrence among the eyes with recurrence. A substantial proportion of recurrences occurred within the first 12 months, whereas another cluster was observed after 24 months, suggesting a possible bimodal tendency (Fig. 2). This temporal heterogeneity may partly reflect differences in disease activity rather than differences in visual prognosis. Early recurrence was associated with a longer axial length, suggesting that eyes with more advanced myopic changes may have a less stable microenvironment, including structural alterations in the RPE/Bruch’s membrane complex [20–22]. In contrast, late recurrence may be related to gradual disease progression or delayed reactivation of previously quiescent lesions.

Our observation of early clustering is consistent with previous studies, which demonstrate that recurrences predominantly occur within the first year after treatment. For example, Yang et al. reported that 72.7% of recurrences occurred during the first year, whereas the post-RADIANCE study showed a markedly lower recurrence rate beyond 12 months than that in the early period [6, 12]. These findings support the notion that the first year is a critical time for recurrence, likely reflecting higher disease activity during the early post-treatment phase. However, our result of temporal distribution should be interpreted with caution because the conservative recurrence definition used in the present study may have delayed the identification of some early recurrence events, leading to an apparent underestimation of early recurrences compared with previous studies.

Despite differences in timing and clinical characteristics, visual outcomes were not significantly influenced by recurrence timing in eyes with recurrence. Both groups demonstrated similar BCVA trajectories over time, without significant differences at recurrence or during follow-up after retreatment. These findings suggest that, among eyes with recurrent myopic CNV, no association between recurrence timing and visual prognosis was detected in this cohort when appropriate retreatment was provided.

Importantly, baseline BCVA was the only factor significantly associated with final BCVA in our study. Previous studies have suggested that not only baseline BCVA but also recurrence itself may influence long-term visual outcomes in myopic CNV [12, 17]. However, in the present study, the analyses were restricted to eyes that had already developed recurrence, thereby limiting the ability to evaluate recurrence as an independent prognostic factor. In this context, baseline BCVA emerged as the strongest factor associated with final visual outcome, consistent with prior evidence that consistently identifies baseline BCVA as the strongest predictor of long-term prognosis in eyes with myopic CNV.

However, no significant associations were detected between final BCVA and recurrence timing, axial length, or baseline IRF. Additionally, exploratory analyses treating time to recurrence as continuous variable yielded results consistent with those of the primary dichotomized analysis, showing no significant association between time to recurrence and final BCVA. These findings suggest that, among eyes with recurrence, the timing of recurrence itself was not associated with long-term visual outcomes.

The results remained consistent after additional adjustment for total follow-up duration. Nevertheless, residual bias related to unequal observation periods cannot be completely excluded because final BCVA was assessed at the last follow-up visit and the total follow-up duration differed between the groups. Therefore, these findings should be interpreted with caution.

We also observed differences in structural features at recurrence. The late recurrence group had greater SHRM height, which may reflect more chronic or organized lesion characteristics. Previous studies have suggested that structural abnormalities on OCT are associated with disease activity and recurrence risk [23, 24]. Although fibrosis after recurrence was observed only in the late recurrence group, the difference was not statistically significant, suggesting that the greater SHRM height cannot be explained solely by fibrotic change. Rather, it may reflect broader structural remodeling associated with chronic lesion evolution. Despite these structural differences, visual outcomes remained comparable between the groups, further supporting the concept that recurrence timing may reflect differences in disease behavior rather than visual prognosis.

This study has some limitations. First, its retrospective design introduces the potential for selection bias. Second, the relatively small sample size may have limited the ability to detect subtle differences. Moreover, because the multivariable model included several covariates despite the limited number of recurrent eyes, the multivariable findings should be considered exploratory and interpreted with caution. Third, the late recurrence group had a significantly longer follow-up duration than the early recurrence group. Because final BCVA was assessed at the last available follow-up visit rather than at a standardized time point, differences in observation time may have influenced visual outcome comparisons. Although total follow-up duration was included in the multivariable model, residual bias related to unequal observation periods cannot be completely excluded. Fourth, all eyes were initially treated using a three-loading-dose followed by PRN regimen. Previous studies have reported comparable visual outcomes between 1 + PRN and 3 + PRN strategies, although several studies have suggested a longer interval to retreatment with the 3 + PRN regimen [11, 25–28]. Therefore, the treatment protocol used in this study may have influenced recurrence timing, and caution is warranted when extrapolating these findings to cohorts managed with a 1 + PRN strategy. In addition, because retreatment decisions were made in routine clinical practice by multiple retina specialists, variability in retreatment thresholds may have influenced the timing at which recurrence was documented. Fifth, because the recurrent-eye cohort was predominantly composed of bevacizumab-treated eyes (84.4%), with only a small number of ranibizumab-treated eyes and no aflibercept-treated eyes, the potential impact of anti-VEGF agent selection on recurrence timing could not be adequately evaluated. Therefore, the generalizability of these findings across different anti-VEGF agents should be interpreted with caution.

In conclusion, among eyes with recurrent myopic CNV in this retrospective cohort, no significant visual difference was detected between the early and late recurrence groups despite differences in certain clinical and structural characteristics. Baseline BCVA was the main factor associated with long-term visual outcome, whereas no association between recurrence timing and visual outcomes was detected in this cohort. However, the relatively small sample size limits the ability to exclude a subtle prognostic effect of recurrence timing. Larger prospective studies are warranted to validate these findings.

## Data Availability

No datasets were generated or analysed during the current study.
